# Strength deterioration mechanism of bentonite modified loess after wetting–drying cycles

**DOI:** 10.1038/s41598-022-06962-6

**Published:** 2022-02-24

**Authors:** Ze-Lin Niu, Jian Xu, Yan-Feng Li, Ze-Feng Wang, Bao Wang

**Affiliations:** 1 School of Civil Engineering, Xi’an University of Architecture and Technology, Xi’an, 710055 Shaanxi China; 2 Shaanxi Key Laboratory of Geotechnical and Underground Space Engineering, Xi’an University of Architecture and Technology, Xi’an, 710055 Shaanxi China; 3 School of Environmental and Municipal Engineering, Xi’an University of Architecture and Technology, Xi’an, 710055 Shaanxi China

**Keywords:** Civil engineering, Natural hazards

## Abstract

The employment of bentonite modified loess (BML) is a common method of constructing the anti-seepage lining of landfills in the loess region of China, and its long-term secure performance is threatened by wetting–drying (W–D) cycles. Taking the remolded loess (RL) and BML with 15% in mass of bentonite as research objects, the W–D cycles test, scanning electron microscope test and direct shear test were carried out to analyze the effects of W–D cycles on the microstructure and shear strength of samples. The regression equations between strength and micro-pore structure parameters were established by the multivariate linear stepwise regression method. The damage mechanism of BML after W–D cycles was studied by establishing damage degree models based on pore area ratio and cohesion. Results indicate that the water absorption and expansion of bentonite effectively block the intergranular pores, resulting in more medium and small pores and more pronounced surface contact of particles. After W–D cycles, the particle arrangement of samples before and after bentonite modification tends to be loose. Both the pore area ratio and fractal dimension increase and tend to stabilize after five cycles. The BML exhibits lower pore area ratio and greater fractal dimension while its cohesion and internal friction angle show more significant decrease after W–D cycles than those of RL. The damage variables based on pore area ratio and cohesion well describe the W–D induced damage of loess before and after modification from macro- and micro-scale perspectives. The damage degree of samples increases with W–D cycles, but the increment decreases.

## Introduction

There are abundant loess resources in the Northwest of China, which refers to silt deposits formed by wind transport in the Quaternary period. Loess interparticle calcite and clay minerals cementation and silt particle morphology make the soil show a metastable structure^1^. After compacted loess is immersed in water, the dissolution of calcite and hydration swelling of clay minerals will weaken the cementation of the soil-aggregate cementation structure, resulting in a reduction of strength and bearing capacity of the loess^2^. The weak cementation between loess particles results in large pores and high porosity. Sanitary landfill is the primary disposal method of municipal solid waste in the Northwest of China, and the compacted loess liner is a good choice for the anti-seepage system at the bottom of the landfill. However, the loess as an anti-seepage layer at the landfill bottom does not meet the requirements of permeability and strength. Therefore, how to modify the loess to suit compacted clay liner requirements has become one of the research hotspots.

Bentonite has large specific surface area and strong cation exchange, which makes it have good hygroscopic expansibility and low permeability. When added into soil as modifier, it can effectively improve the soil microstructure and enhance the strength of soil^3,4^. Scholars have carried out relevant research on bentonite modified soil from the aspects of microstructure and macro strength. Through scanning electron microscope (SEM) test, the particle arrangement and pore structure of bentonite modified soil sample can be analyzed intuitively from two-dimensional aspect^5^, while the mercury intrusion porosimetry (MIP) method can analyze the overall pore size distribution characteristics of the sample from three-dimensional perspective^6^. With the increase of bentonite content in loess, the number of pores increased dramatically, but the pore area and pore diameter show a downward trend^7^. Nano-bentonite can promote the internal cementation of clay particles and effectively reduce the compressibility of clay^8^. The hydration, agglomeration and interparticle flocculation caused by additives such as bentonite and lime in loess promote the intergranular cementation and change the plasticity of the modified loess^9^. The bentonite content had a considerable effect on the cohesion of bentonite modified loess (BML), but insignificant on the internal friction angle^10^. Low content of hydrophilic nano bentonite reduces the expansion potential of expansive soil and increases the strength, and it is feasible to apply the modified clay to the landfill cover^11^. The macro characteristics such as compressibility, rheological properties and strength of bentonite modified soil are the reflection of its microstructure properties, while the long-term durability of bentonite modified soil under natural environment needs to be further studied.

The Loess Plateau of China is a typical arid and semi-arid region, where the loess is subject to frequent wetting–drying (W–D) cycles due to rainfall and evaporation^12^. Therefore, it is necessary to study the influence of W–D cycles on the strength and microstructure of loess. The formation of cracks in loess is closely related to the matric suction change and the decrease of loess strength^13–16^. Scholars have studied the microstructure of loess under W–D cycles through multiscale methods^17,18^. The seepage process of silty loess will reduce the pore ion concentration and the bonding strength of clay minerals, make smaller silt particles fall off from the aggregates, and weaken the soil structure^19^. The change of hydraulic state during the W–D cycles is accompanied by the change of microstructure, mainly played by the pores between particles and aggregates and the pores inside clay aggregates^20^. The change of loess saturation caused by W–D cycles is the main factor leading to soil cracking. Ye et al.^21^ used SEM and nuclear magnetic resonance (NMR) technology to study the microscopic characteristics of paleosols, found that the proportion of micropores decreases after W–D cycles while those of the macropores and mesopores increase. Xu et al.^22,23^ studied the failure mechanism of sodium sulfate saline intact loess under the coupled action of W–D cycles and salt weathering. The lime and fly ash modified loess under W–D cycles has also attracted wide attention^24,25^. Yan et al.^24^ studied the unconfined compressive strength and pore size distribution of lime-fly ash loess (LFL) under W–D and freeze–thaw conditions. Zhang et al.^25^ studied the influence of W–D cycles on the strength of cement stabilized loess (CSL). However, there are few studies on the relationship between the mechanical properties and microstructure of BML under W–D cycles. The degradation mechanism of strength and microstructure of BML under W–D cycles needs to be further studied.

In this study, laboratory tests were carried out on the remolded loess (RL) and BML with 15% in mass of bentonite to investigate the shear strength and microstructure characteristics after W–D cycles and to explore the macro-and micro-scale damage mechanism under W–D cycles and put forward damage models of BML after W–D cycles. The results will have a particular reference value for improving local loess resources in Northwest China and the construction of anti-seepage linings in landfills.

## Materials and methods

### Soil property

**Figure 1:**
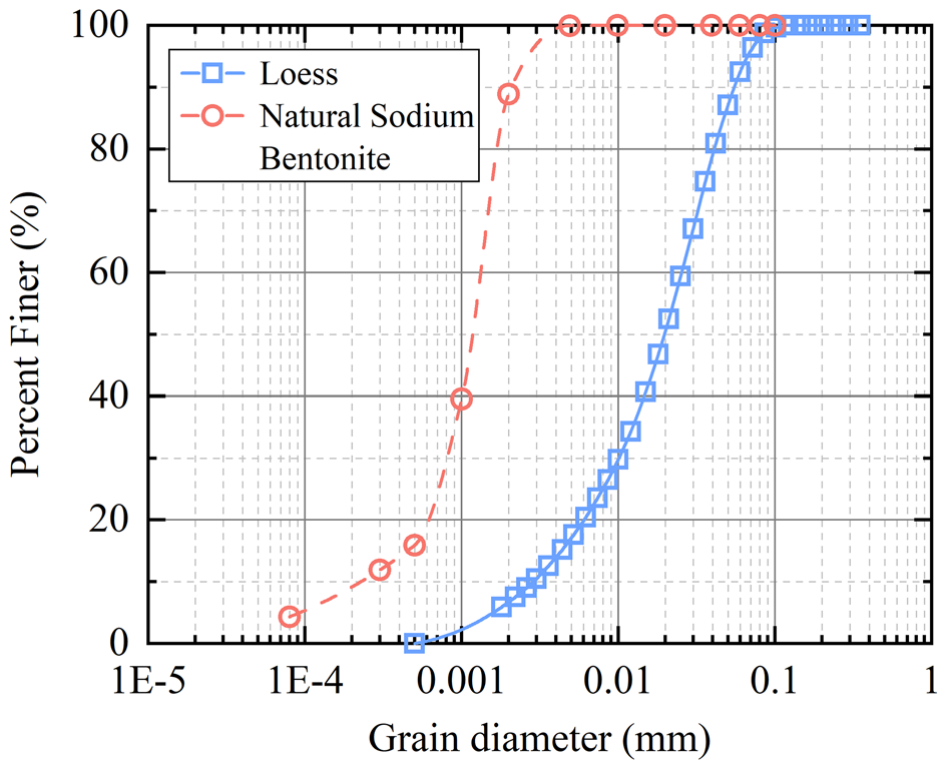
The particle-size distribution curve of test soil.

The test loess was taken from the sidewall of a foundation pit in Xi'an, Shaanxi Province, with a depth of 2–3 m from the surface. It belongs to the late Pleistocene Q_3_ loess with yellowish-brown color and obvious vertical joints. The loess samples were pre-treated sufficiently by air-drying, grinding, and sieving. The Atterberg limits (ASTM D4318-17e1)^26^ and compaction characteristics (ASTM D0698-12R21)^27^ of the loess samples were analyzed, as listed in Table 1. The cohesion and internal friction angle of the loess were 58.68 kPa and 21.42° obtained by direct shear test, respectively. The particle size distribution curve of test loess is shown in Fig. 1. The uniformity coefficient *Cu* is 8.33, and the curvature coefficient *Cc* is 1.33. The distribution of grain composition of the loess is: sand (12.88%), silt (80.42%), and clay (6.7%). The bentonite used in the tests is natural sodium-based bentonite, which is grey-white and powdery. The basic properties such as Atterberg limit (ASTM D4318-17e1)^26^, swelling index (ASTM D5890-19)^28^ and Anion exchange capacity (ASTM D7503-18)^29^ were determined as shown in Table 2. The basic properties of bentonite are the particle size distribution curve is shown in Fig. 1. The *Cu* is 5.65, and *Cc* is 2.19.

**Table 1:** Physical properties of the loess.

Liquid limit, *w*_*L*_ (%)	Plastic limit, *w*_*P*_ (%)	Plasticity index, *I*_p_ (%)	Initial water content, *w* (%)	Optimum moisture content, *w*_op_ (%)	Maximum dry density *ρ*_dmax_ (g/cm^3^)	Cohesion, *c* (kPa)	Internal friction angle, *φ* (°)
30.2	17.8	12.4	22.32	18	1.54	58.68	21.42

**Table 2:** Physical properties of the sodium bentonite.

Specific gravity *G*_s_	Liquid limit, *w*_*L*_ (%)	Plastic limit, *w*_*P*_ (%)	Plasticity index, *I*_p_ (%)	Swelling index (mL/2 g)	Anion exchange capacity (meq/100 g)
2.65	314	40	274	39	91

### Preparation of soil samples

**Figure 2:**
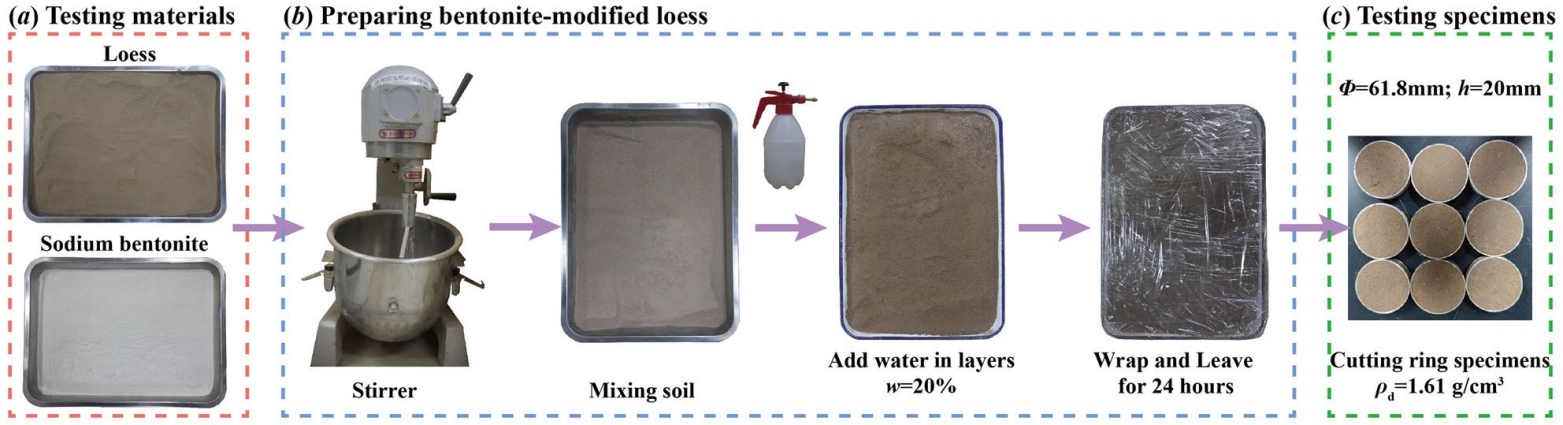
The preparation process of bentonite modified loess.

BML samples were prepared according to the percentage of the dry weight of bentonite to that of loess. The calculated loess and bentonite were poured into a stirrer (variable frequency spend-adjusting slurry stirrer) to mix and homogenize sufficiently. Through the standard compaction test, the optimal bentonite content is 15%. The optimal water content of 15% BML is 20%, and the maximum dry density is 1.61 g/cm^3^. The water content and dry density of the improved loess samples are set accordingly. The direct shear test samples of RL and BML are made of ring knife samples with a diameter of 61.8 mm and a height of 20 mm. The SEM samples are cut from the ring knife samples with a size of 10 mm × 10 mm × 20 mm, and the preparation process of BML is shown in Fig. 2.

### Tests procedures

#### Wetting–drying cycles

**Figure 3:**
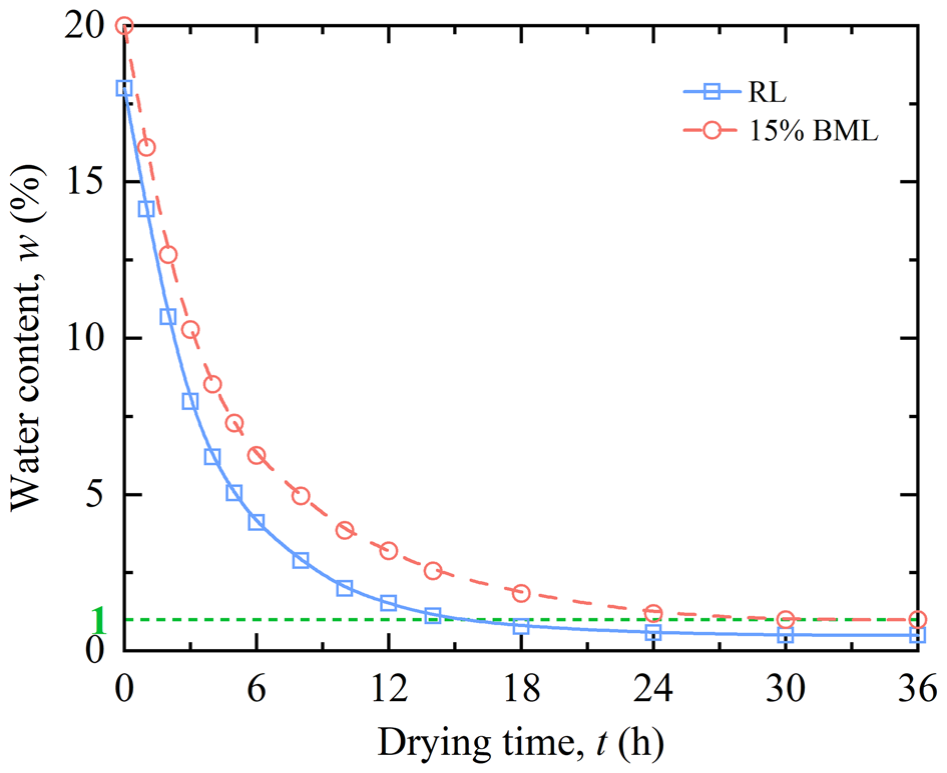
Relationship between water content and drying time.

The drying process of samples adopts an electric thermostatic drying oven. The temperature control range is + 10–250 °C, and the temperature fluctuation is ± 1 °C. During the test, to simulate the process of wetting–drying cycles on the land surface, the highest temperature in summer in loess region was used as the drying temperature, and to increase the drying rate and reduce the influence of temperature on the structure of soil samples, the drying temperature was set to 45 °C. The time required for the water content of the samples to reach a stable state was verified by trial tests. The test procedure is shown as follows. Two sets of standard ring knife samples (*d* = 61.8 mm, *h* = 20 mm) of RL and 15% BML were weighed and placed in a drying oven for drying at 45 °C, taken out at regular intervals and weighed to calculate the water content. Figure 3 shows the relationship between water content and the drying time. The water content of the samples decreases over drying time. The water content of the samples decreases significantly in the first 6 h and tends to be stable after 24 h. Besides, the water content of RL decreases over drying time, which is higher than that of the BML. The water content of RL is more diminutive than BML after reaching a stable state, but the water content is less than 1%. Therefore, it can be seen from the above verification test that the samples drying at 45 °C for 24 h can achieve a better drying effect, and the drying time is set to 24 h.

The water film transfer method is used in the sample wetting process. The filter paper is more prominent than the sample diameter and is placed on the surface of the sample. A certain amount of water is added to the filter paper through the dropper. The water evenly penetrated the soil through the filter paper. When the water content reaches the preset value, the samples are fully wrapped with a plastic film and kept in a close chamber for 24 h. The water is evenly diffused in the soil to complete a W–D cycle. The number of W–D cycles is 0, 1, 2, 3, 4, 5, and 10.

#### Direct shear test

After different W–D cycles, the samples were mounted on the strain-controlled direct shear test apparatus. Considering the actual landfill load, the normal pressure is set to 30, 50, 100, 150 kPa, respectively, which represents the change of normal pressure at the top of the impervious layer during the whole landfill process. The shear rate of the test is 0.08 mm/min. If the shear stress has a peak, continue to shear until the shear displacement reaches 4 mm or stop the test when the shear displacement reaches 6 mm.

#### SEM test

**Figure 4:**
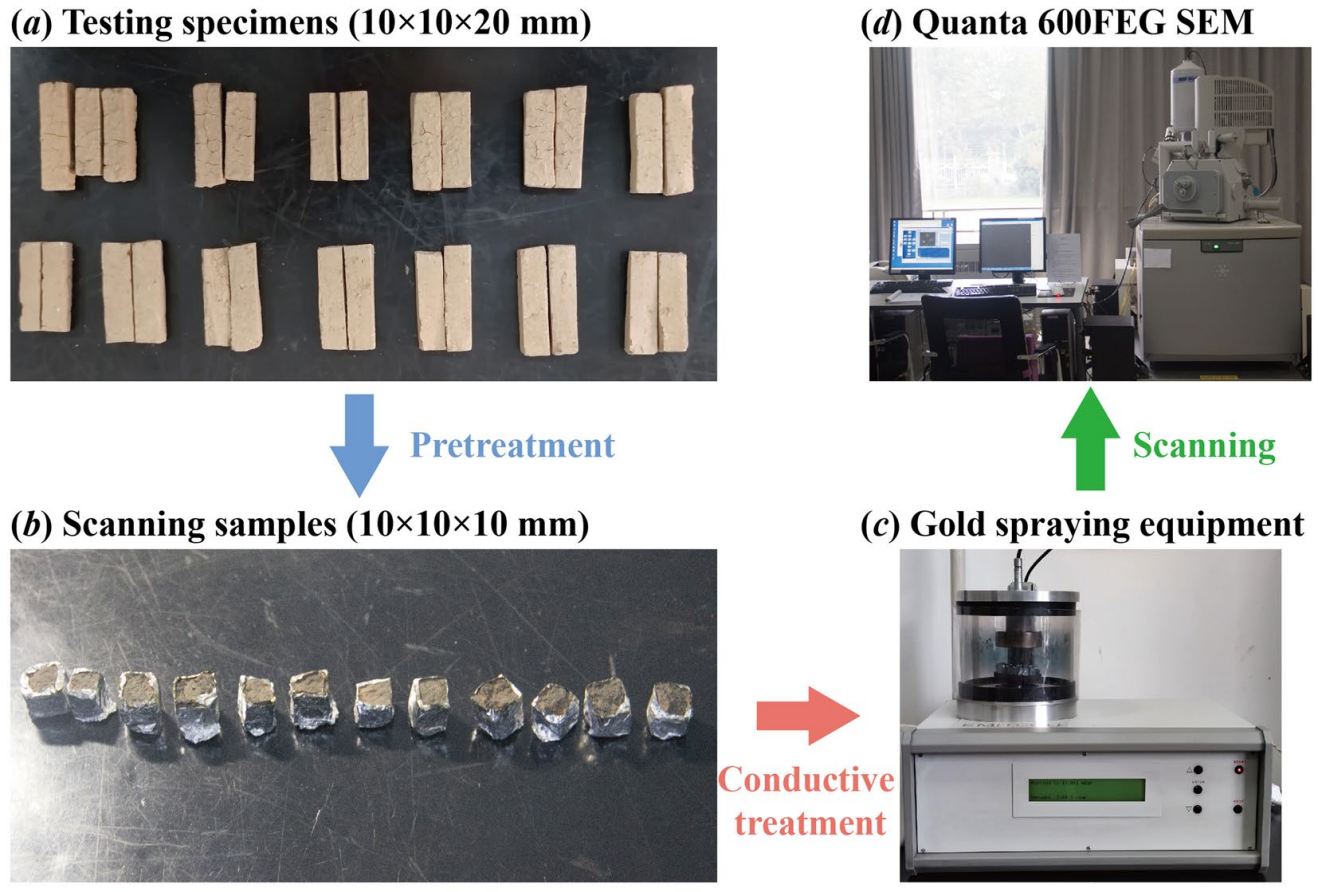
SEM test procedure.

The test process for the SEM tests is shown in Fig. 4. The SEM samples after different W–D cycles were broken from the middle, and the flat section without obvious cracks was selected as the observation surface. The sidewall of the selected sample was wrapped tightly with tin foil to expose the observation section, which was then fixed on the metal base with conductive glue. Then gold plating was performed to make the surface of the sample conductive after vacuuming and then used in SEM scanning. The Quanta 600FEG SEM was used in the SEM tests. Three points are selected on each sample surface to ensure that representative structural units can be thoroughly selected. The 1000 and 2000 magnification are selected to scan the sample, respectively.

## Results

### Microstructure of loess after W–D cycles

#### Qualitative analysis of SEM images of samples

**Figure 5:**
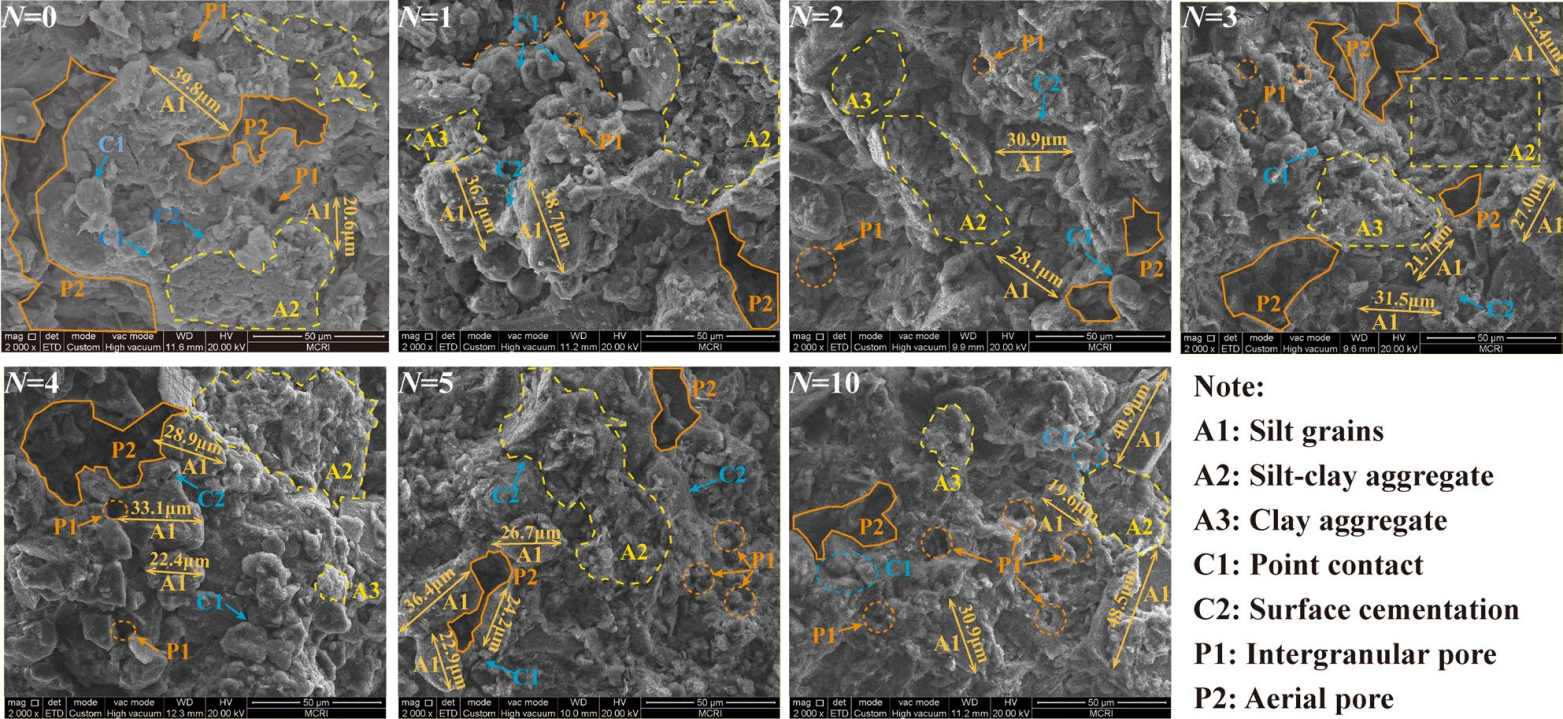
SEM images of RL under W–D cycles (*N*) (× 2000).

Figure 5 presents the SEM images (× 2000) of the RL after different W–D cycles. From the Figure, the soil particles of the RL without W–D cycles are mainly silt grains (A1) and silt–clay aggregates (A2), and the contact mode is mainly point contact (C1) and surface cementation (C2). The aerial pores (P2) distribution has an obvious difference with the intergranular pores (P1), the aerial pores are inter-connective between soil particles. The skeletal structure of the loess presents a clot mosaic colloidal structure. With the increase of W–D cycles, the clay films are generated on the surface of silt grains (A1) with a diameter of about 30 μm. The partially cemented silt–clay aggregates (A2) gradually separate during the water migration process, the number of intergranular pores increases and some intergranular pores are gradually connected to form new water migration channels. The increased pores lead to a decrease in matric suction and capillary pressure, which results in the reduction of loess strength^30^. After four W–D cycles, the aerial pores (P2) continue to serve as the main water migration channel, and partial clay minerals dissolution results in weakened particles bonding, leading to an increase in the number of intergranular pores (P1), and the soil structure is further weakened. However, after five W–D cycles, the changes in the number of pores and soil particles are insignificant, and a new relatively stable structure was formed inside the soil, and the soil strength stabilized with the increase of W–D cycles.

**Figure 6:**
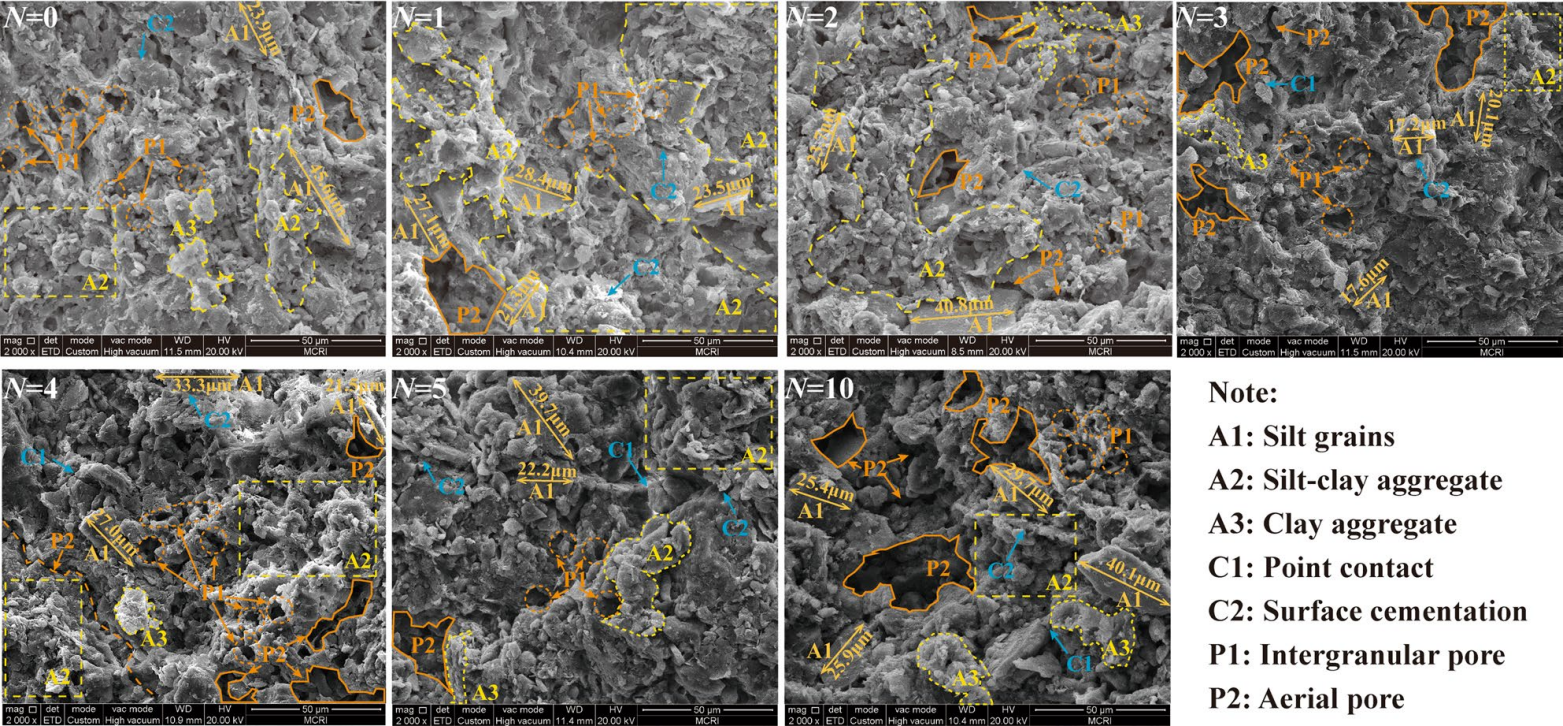
SEM images of BML under W–D cycles (*N*) (× 2000).

Figure 6 shows the SEM images (×2000) of BML with different W–D cycles. From the figure, the particles of BML without the W–D cycle are mainly composed of cemented clay aggregates (A3), the flocculated minerals wrapped around the loess particles and filled in between the pores of the particles. The contact between particles is mainly surface cementation (C2), and the pores are mainly small and medium-sized intergranular pores (P1). The skeleton of the modified loess particles presents an inlaid cemented structure, so the structure of the modified loess is more stable than unmodified loess. The cementation between the soil particles weakens after the fourth W–D cycle, but the contact between particles is still dominated by surface cementation (C2), the soil structure becomes looser, and intergranular pores (P1) with larger diameters are generated. After five W–D cycles, the contact mode between particles gradually transitions from surface cementation (C2) to point contact (C1). The subdividing of bentonite particles produces irreversible damage to the microstructure of modified loess^31^. The small and medium-sized pores continue to decrease, while the intergranular pores (P1) gradually increase and tend to be connected. The pore wall develops smoothly as a water migration channel during W–D cycles. After the tenth W–D cycle, the monomer silt particles with a diameter of about 25 μm in the skeleton particles increases, the cemented clay minerals are significantly reduced, the cementation between the particles is weak, and the contact mode is mainly point contact (C1). The pores are mainly aerial pores (P2), which leads to the weakening of the soil structure and strength.

#### Quantitative analysis of loess SEM images

**Figure 7:**
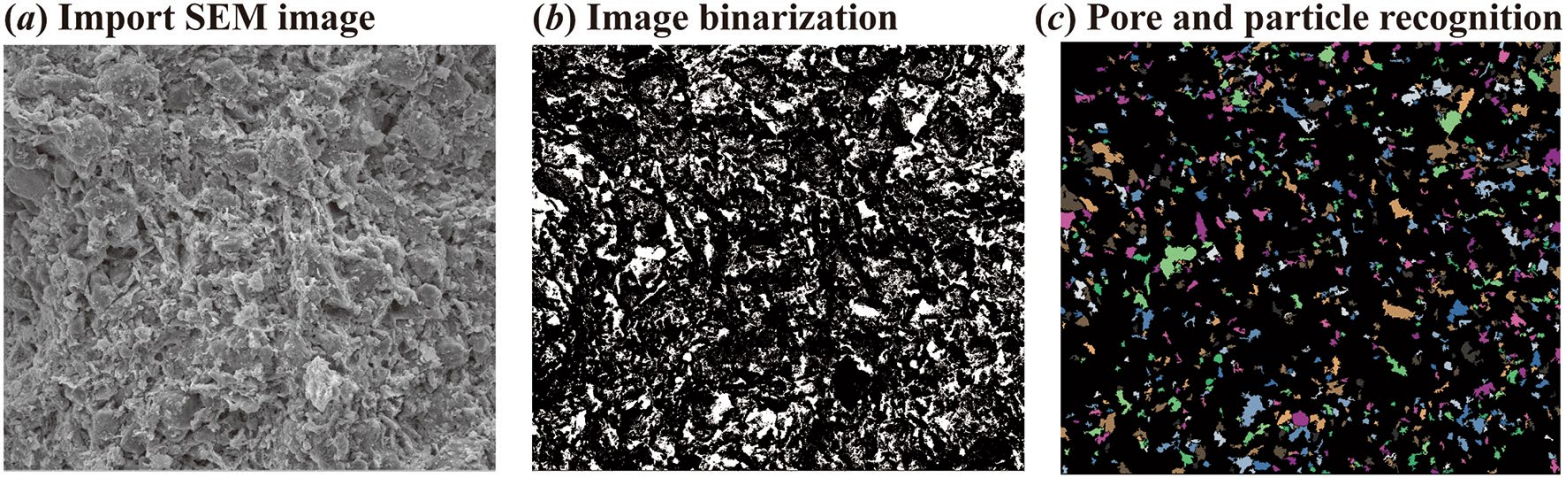
Pores and particles identification and analysis process by PCAS system.

Based on the particles (pores) and cracks analysis system (PCAS)^32^, SEM images with the magnification of 1000 times were selected for microscopic quantitative analysis. Figure 7 shows the identification and analysis process of pores and particles. Binarized images are obtained through threshold segmentation and denoising steps, and the pores and particles are automatically segmented and identified^33^. The white and black areas represent the pores and particles, respectively. Various geometric parameters and statistical parameters of pores and particles are obtained by a built-in algorithm. The quantitative indicators of microstructure such as pore area ratio (*n*) and fractal dimension (*D*_*f*_) were selected to describe the changes of particles and pores of the samples after the W–D cycles, which can be described by:

1 $$n = \frac{{A_{v} }}{A}$$

2 $$\log \left( C \right) = \left( {D_{f} /2} \right) \cdot \log \left( S \right) + c_{1}$$

where *A*_v_ and *A* are the pore area and the total area in the statistical area; *C* and *S* are the perimeter and area of the pore; *c*_1_ is a constant.

The fractal dimension of the pore reflects the development and complexity of the soil. Generally, the larger the fractal dimension, the more complex the pore structure distribution, the scattered distribution of soil particles and the weak degree of agglomeration.

**Figure 8:**
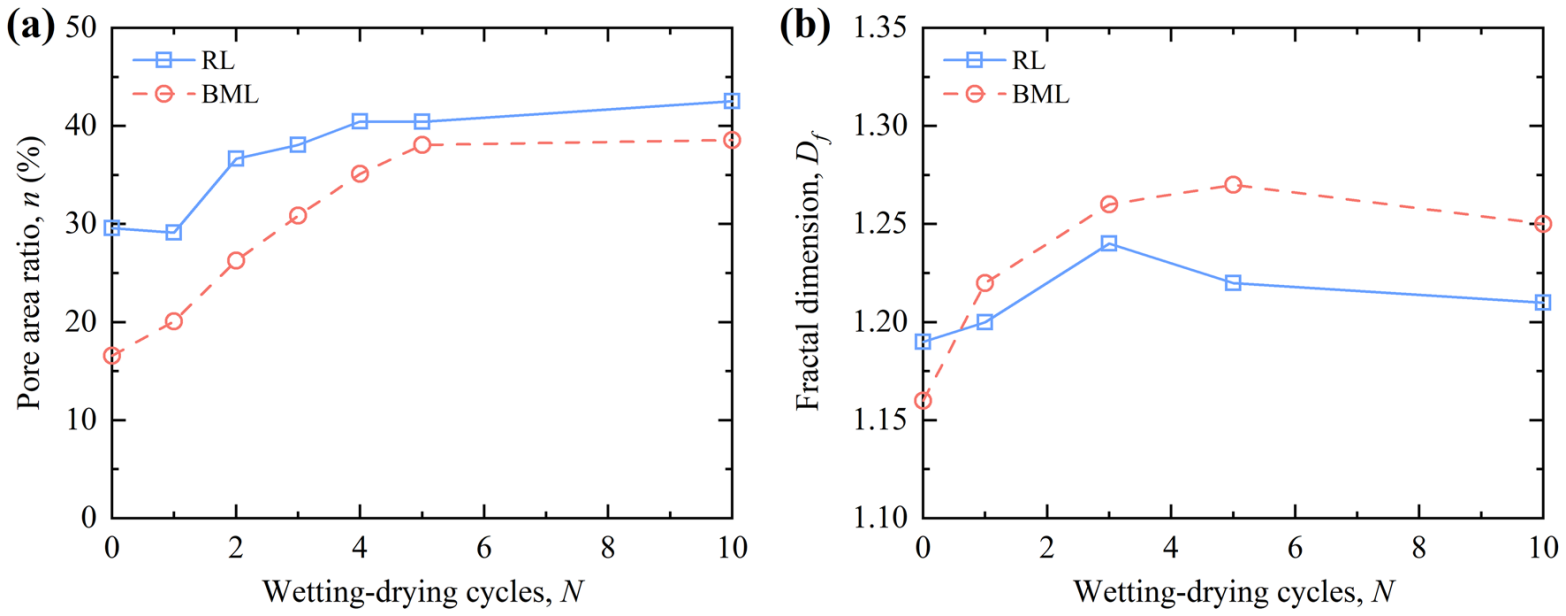
Variation of pore area ratio (*n*) and fractal dimension of loess under W–D cycles.

Figure 8a illustrates the pore area ratio (*n*) of RL and BML after W–D cycles. It can be seen that the *n* of RL and BML increases with the number of W–D cycles and tends to be stable after five cycles. After five W–D cycles, due to the continuous migration of water in the pore channel, some small particles are washed away from the pore wall, the original small and medium-sized pores are enlarged to form larger pores, resulting in higher *n*. After more W–D cycles, the pores are gradually dominated by aerial pores. The pore channels for the migration of water are redistributed and soil particles are rearranged. The original structure becomes a relatively uniform loose structure, and the *n* no longer changes. Furthermore, the *n* of RL is always greater than that of BML after W–D cycles. The pore structure of RL is mainly composed of large intergranular pores between particles. Part of the intergranular pores of loess particles are filled by bentonite particles, which reduces the pore volume of modified loess, while the water absorption and expansion of bentonite effectively blocks the intergranular pores, thus the *n* of BML is lower than that of RL during the whole W–D cycles.

The variations of pore fractal dimension of RL and BML with W–D cycles are shown in Fig. 8b. The results show that the pore fractal dimensions of both the RL and the BML increase first, and tend to be stable after five W–D cycles. The fractal dimension of pores of BML is smaller than that of RL before W–D cycles because of the coexistence of aerial pores and intergranular pores in the pore structure of RL and the uneven distribution of pores among particles. While the filling of bentonite makes the number of large pores in BML decrease sharply, the pores are mainly medium and small, and the pore structure of BML is more complex than that of RL. Therefore, the fractal dimension of RL in the initial state is more significant than that of the improved loess. After W–D cycles, the primary water migration channel in RL is the well-connected pores. While for the BML, the interconnected pore channel is less developed. Influenced by water migration, more small and complex pores are formed in the soil. The number and shape of pores in the soil gradually develop. The pore structure is more complex than that of RL, so the pore fractal dimension is larger than that of RL. After more W–D cycles, due to the rearrangement of soil particles and pores, the soil structure of RL and BML changes from the original mosaic cementation structure to a uniform loose structure, the pore structure is relatively stable, and the fractal dimension of pores tends to be stable after a decreasing trend.

#### Analysis of pore area ratio-based damage degree

To further analyze the change of pore area ratio of RL and BML after W–D cycles, a continuous variable *D* is introduced as the pore area ratio damage degree, as shown in Eq. (3):

3 $$ \left\{ \begin{gathered} D_{1} = \frac{{\left| {n_{1} - n_{01} } \right|}}{{1 - n_{01} }} \hfill \\ D_{2} { = }\frac{{\left| {n_{2} - n_{02} } \right|}}{{1 - n_{02} }} \hfill \\ \end{gathered} \right. $$

where *D*_1_ and *D*_2_ represent the pore area ratio damage of RL and BML after W–D cycles, respectively; *n*_1_ and *n*_2_ are the pore area ratio of RL and BML samples after W–D cycles, respectively (%); *n*_01_ and *n*_02_ are the initial pore area ratio of RL and BML samples (%). *D* = 0 corresponds to the initial undamaged state of the sample, and *D* = 1 indicates that the sample is in a fully damaged state, and 0 < *D* < 1indicates that the sample is in a damaged state.

According to the change curve of pore area ratio in the micro-quantitative index of RL and BML after the W–D cycles in Fig. 8a, it can be found that the relationship between *N* and *n* of RL and BML after different W–D cycles is as follows:

4 $$\left\{ \begin{gathered} n_{1} (N) = a_{1} N + b_{1} N^{2} + n_{01} \hfill \\ n_{2} (N) = a_{2} N + b_{2} N^{2} + n_{02} \hfill \\ \end{gathered} \right.$$

where *N* is the number of W–D cycles; *a*_1_, *a*_2_, *b*_1_, and *b*_2_ are all fitted parameters. Transform the form of Eq. (4) into the following equation:

5 $$\left\{ \begin{gathered} \left| {n_{1} - n_{01} } \right| = \left| {N(a_{1} + b_{1} N)} \right| \hfill \\ \left| {n_{2} - n_{02} } \right| = \left| {N(a_{2} + b_{2} N)} \right| \hfill \\ \end{gathered} \right.$$

Substituting Eq. (5) into Eq. (3) can obtain the univariate prediction model for the number of W–D cycles and pore area ratio damage as follows:

6 $$\left\{ \begin{gathered} D_{1} = \frac{{\left| {N(a_{1} + b_{1} N)} \right|}}{{1 - n_{01} }} \hfill \\ D_{2} = \frac{{\left| {N(a_{2} + b_{2} N)} \right|}}{{1 - n_{02} }} \hfill \\ \end{gathered} \right.$$

where *D*_1_ and *D*_2_ respectively represent the pore area ratio damage of RL and BML after W–D cycles; *N* is the number of W–D cycles; *n*_01_ and *n*_02_ are the initial pore area ratio of RL and BML samples, the values are 29.6% and 16.57%; *a*_1_, *a*_2_, *b*_1_, and *b*_2_ are all fitted parameters, with values of 0.0377, 0.0651, − 0.0023, and − 0.0042 respectively.

In Eq. (6), *D*_1_/*D*_2_ obtains Eq. (7), and which can reflect the relationship between the pore area ratio damage of RL and 15% BML during the W–D cycles:

7 $$\frac{{D_{1} }}{{D_{2} }} = \left| {\frac{{a_{1} + b_{1} N}}{{a_{2} + b_{2} N}}} \right| * \frac{{1 - n_{02} }}{{1 - n_{01} }}$$

**Figure 9:**
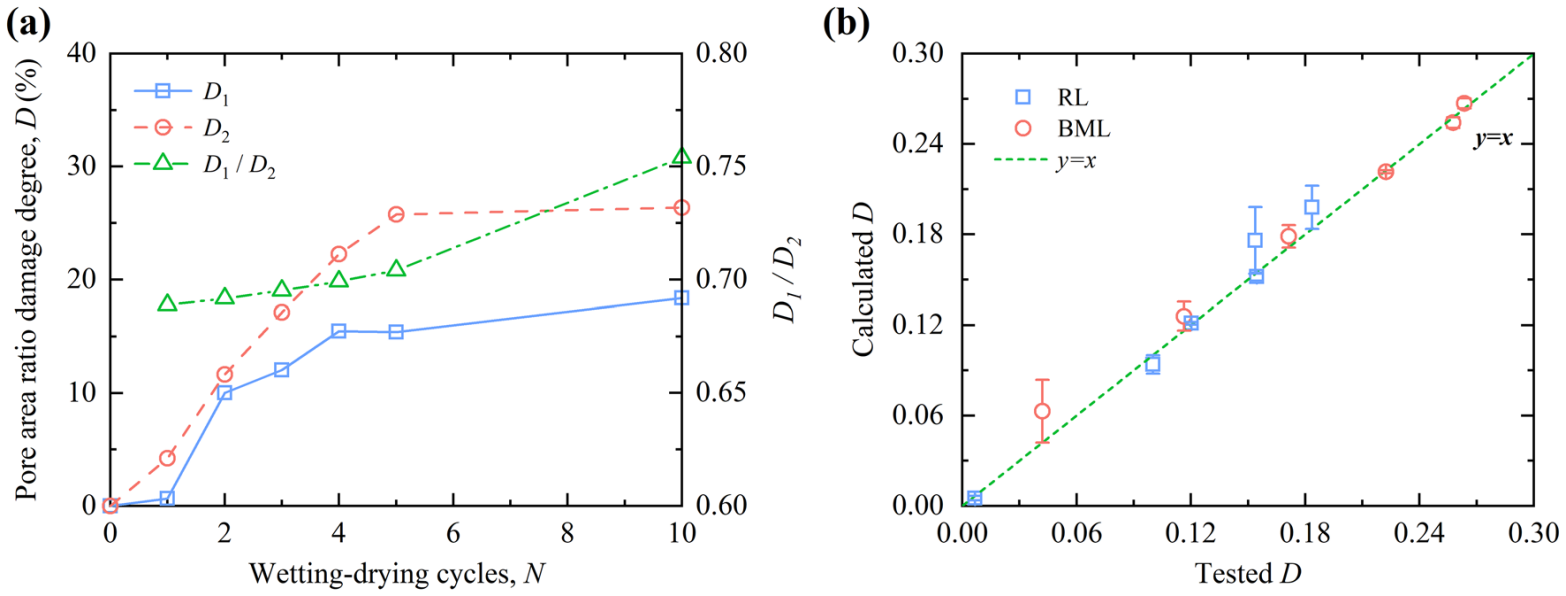
Comparison of pore area ratio damage degree and model results.

Figure 9a presents the variation of the pore area ratio damage degree of RL and BML with the number of W–D cycles. The result shows that the pore area ratio damage of RL and BML increase during the previous five W–D cycles, stabilizing from the fifth W–D cycle. The pore area ratio damage of the BML samples is greater than that of RL during the development of W–D cycles. Although the pore area ratio of RL is always higher than that of BML during the whole W–D cycles, the pore area ratio of BML varies within a large range after the impact of the W–D cycle. The fine bentonite particles swell and fill in the large pores of the loess grains skeleton before the W–D cycle. During the W–D cycles, the montmorillonite continuously undergoes the processes of water-swelling and shrinkage. After several W–D cycles, the damage of the expanded montmorillonite in the process of water shrinkage is irreversible, resulting in a more significant change in the pore structure of the BML than before. Therefore, the pore area ratio damage also shows a larger trend than that of the RL. Figure 9b compares the calculated value and the tested value of the univariate damage prediction model based on the pore area ratio. From the figure that most of the data points are distributed on both sides of the straight-line *y* = *x*, indicating that the predicted value of the damage model is similar to the experimental value, the model can better predict the RL and BML pore area ratio damage rules under the action of W–D cycles.

### Analysis of shear strength of loess under W–D cycles

#### The curves between shear stress and shear displacement

**Figure 10:**
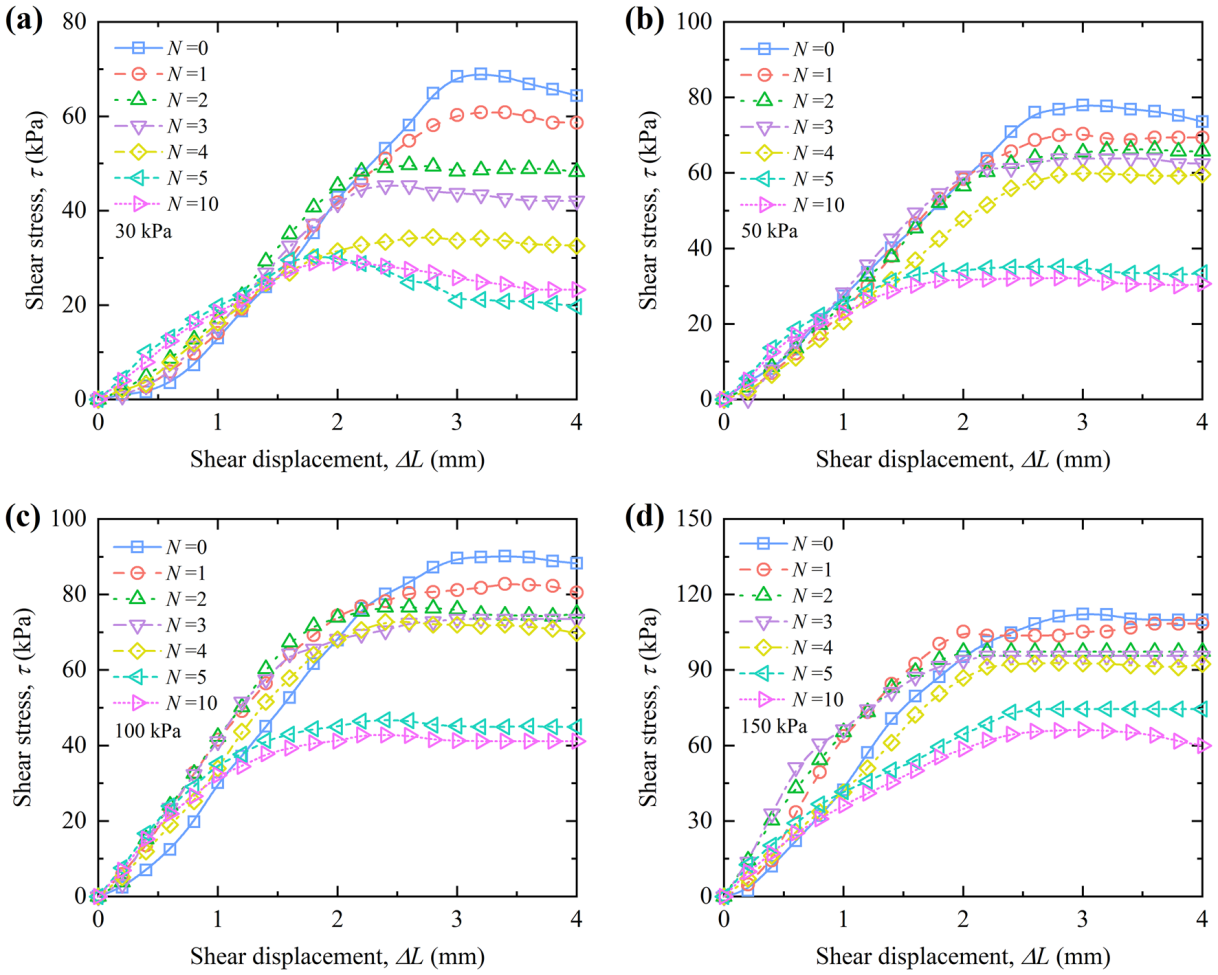
Shear stress versus shear displacement curves of RL under vertical pressure.

**Figure 11:**
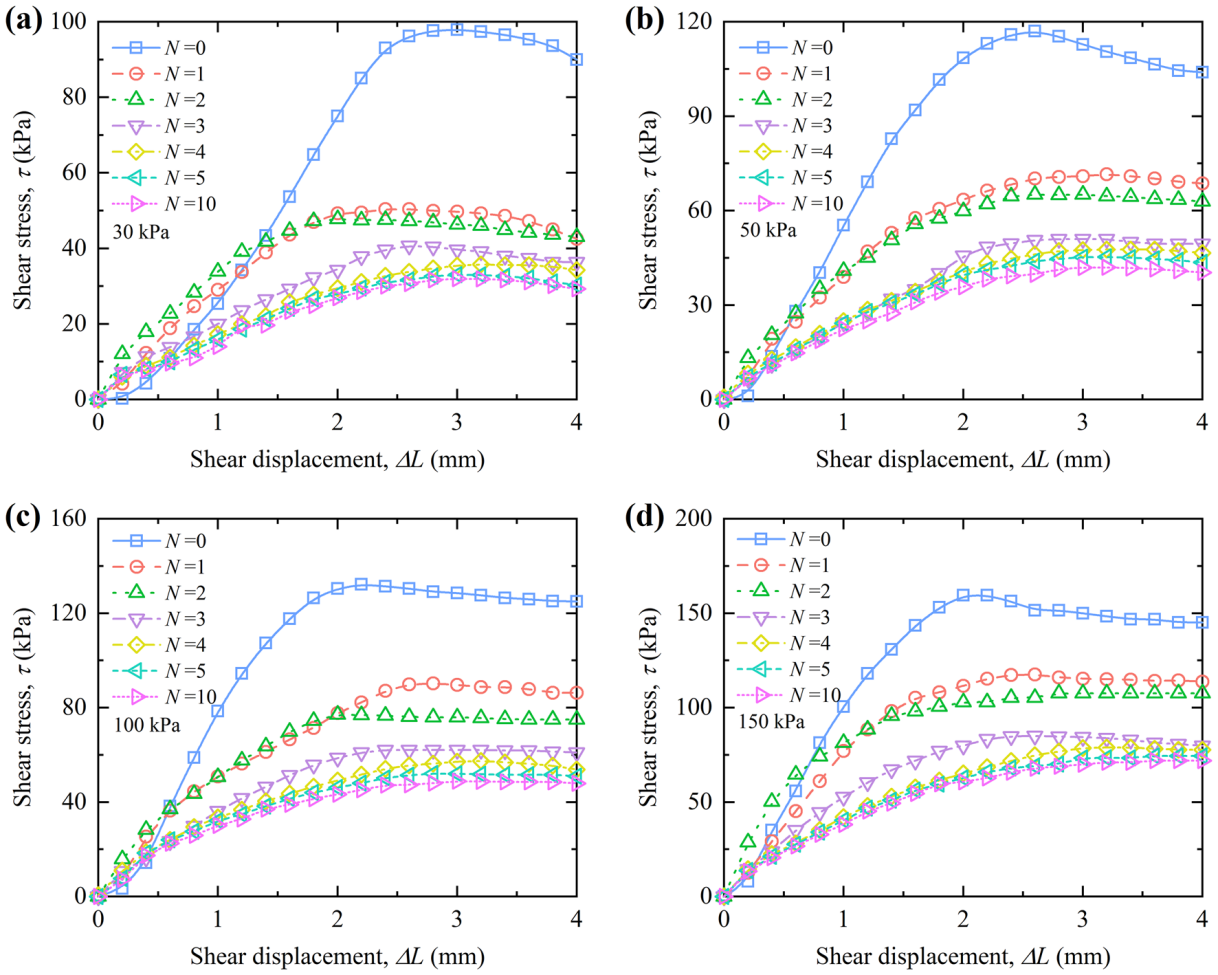
Shear stress versus shear displacement curves of BML under vertical pressure.

Figures 10 and 11 show the relationship between shear stress and shear displacement of RL and BML under different vertical pressures during W–D cycles. The relationship between shear stress and shear displacement of RL shows strain softening under the action of low vertical pressure (*p* = 30 kPa), the curves of RL gradually transits to strain hardening with the vertical pressure increase. The shear stress of samples increases with the growth of shear displacement after different W–D cycles. However, the relationship between shear stress and shear displacement of BML is strain-softening without the influence of W–D cycles, while the relationship between shear stress and shear displacement is strain hardening when it experiences the W–D cycles, and the shear stress increases with the growth of shear displacement.

#### Strength parameters

**Figure 12:**
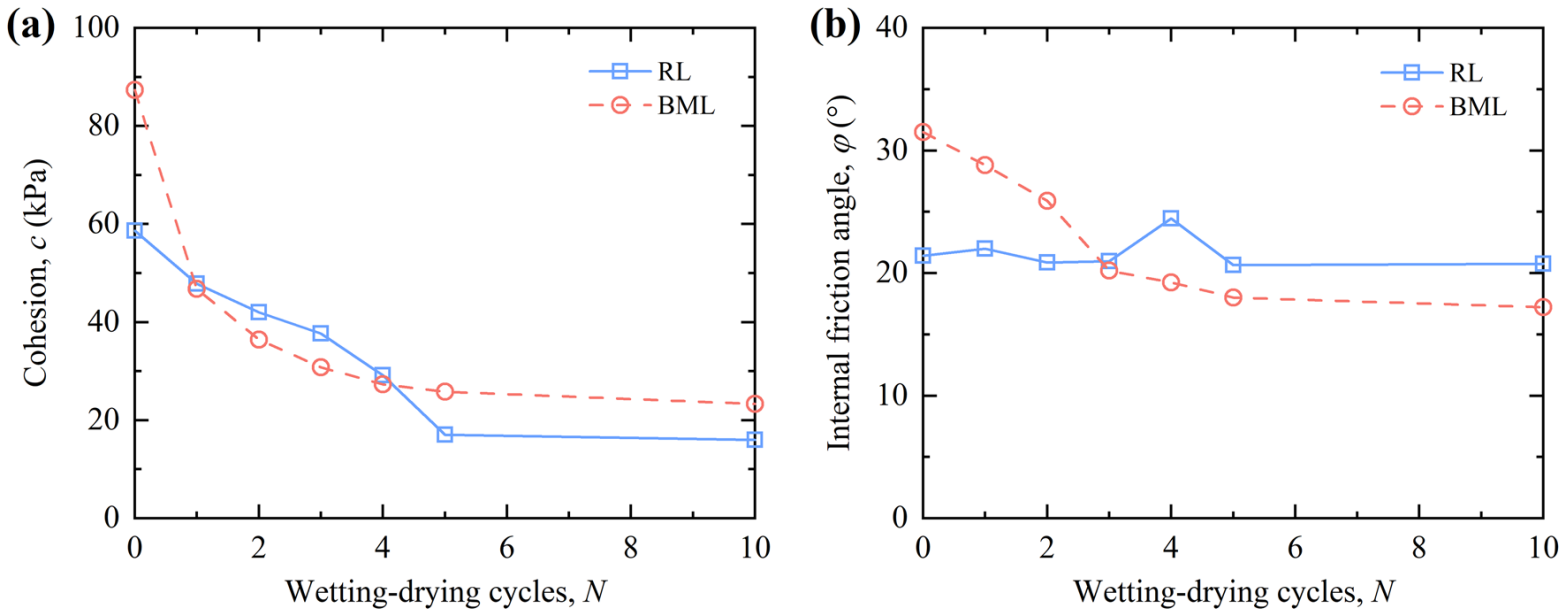
Strength parameters of loess under W–D cycles.

Figure 12a shows the variation of the cohesion of RL and BML with the number of W–D cycles, the cohesion of both types of loess decreased gradually with the increase of the W–D cycles, and tended to be stable after five W–D cycles. The cohesion of BML was higher than that of RL in the initial state, but lower in the first to the fourth W–D cycle. After five cycles, the cohesion of BML is higher than that of RL. The cohesion is related to the cementation between soil particles, double-layer water and the attraction between molecules. Without the effect of the W–D cycle, due to montmorillonite and other minerals in bentonite, water swelling fills the pores between loess particles, and ion exchange forms a diffuse double layer, which enhances the bonding force and electrostatic attraction between soil particles. The interaction force between modified loess particles is more substantial than RL particles, so the modified loess cohesion is greater than RL. During the W–D cycles, the cement structure and diffusion electric double layer are destroyed due to water migration, and the cohesion decreases sharply. Finally, when the cohesion tends to be stable, the existence of some clay aggregates makes the cohesion of modified still higher than that of RL after multiple W–D cycles.

Figure 12b presents the variation curve of the internal friction angle of RL and BML with the number of W–D cycles. As shown in the figure, the internal friction angle of RL samples fluctuates with the increase of the W–D cycles, while the internal friction angle of BML shows a trend of continuous attenuation with the increase of the W–D cycles and the attenuation amplitude gradually decreases. The internal friction angle of the sample depends on the friction and occlusion between the particles. The addition of bentonite in the loess acts as the clay aggregates between the soil particles, and more small particles adsorb on the surface of particles, causing the friction between the soil particles to increase. With the development of W–D cycles, the friction between particles weakens, and the internal friction angle between particles decreases due to the lubrication of water film.

### Analysis of shear strength

**Figure 13:**
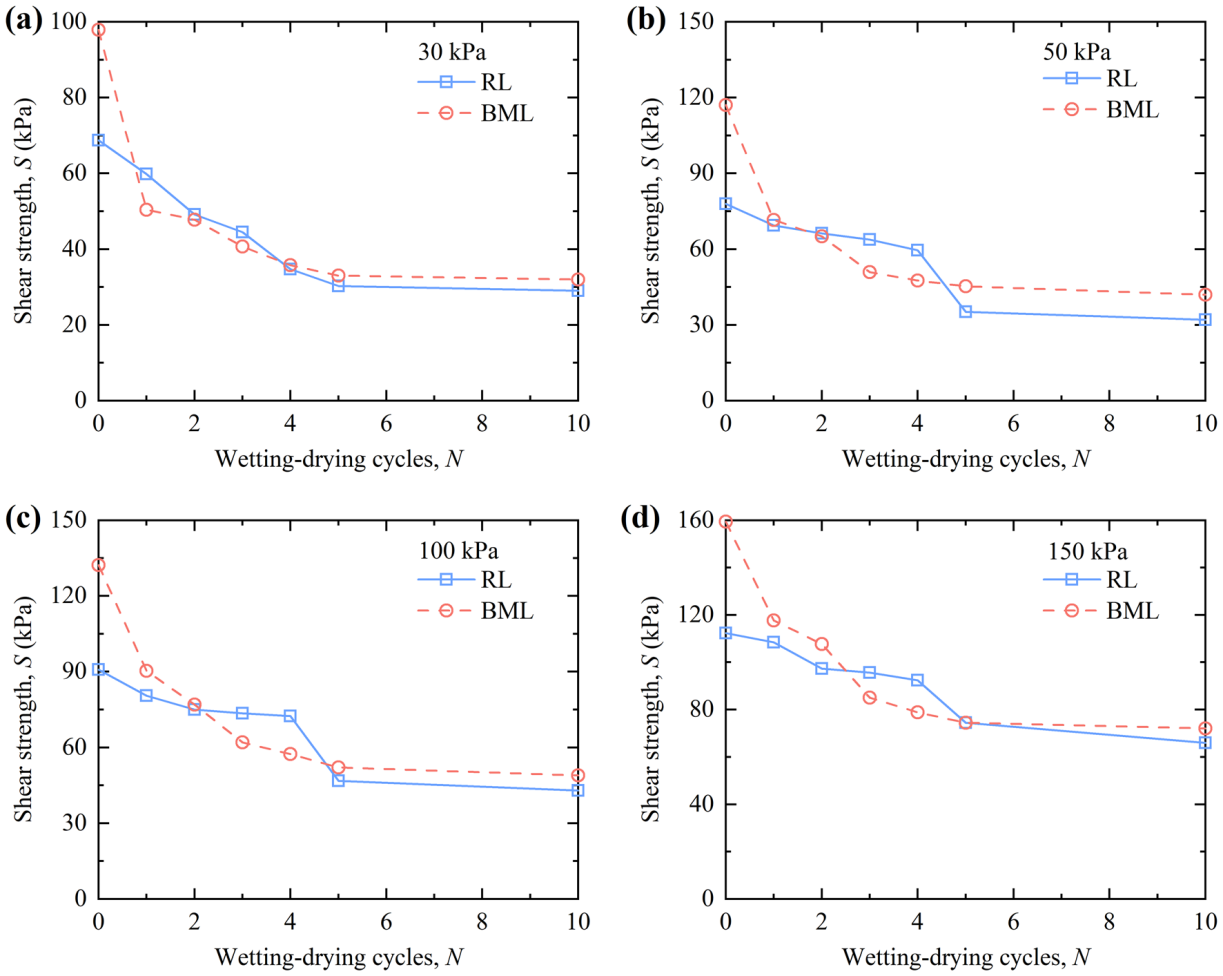
Shear strength curves of loess under W–D cycles.

Figure 13 illustrates the variation of shear strength of RL and BML under different W–D cycles. Under different vertical pressures, the shear strength of RL and BML first decreases and then tends to be stable with the increase of the number of W–D cycles, which is consistent with the variation law of cohesion under W–D cycles. Before the W–D cycles, the clay minerals in the BML fill in the aerial pores and large intergranular voids, the soil particles are mainly surface cementation, the BML particles structural stability is higher than that of the RL, so the shear strength of the BML is higher than that of the RL. The shear strength of BML is lower than that of RL after the W–D cycles, but the shear strength of BML and RL tends to be stable after five W–D cycles, and the shear strength of BML is higher than that of RL. In the previous W–D cycles, as the scouring effect of water migration on soil particles, the clay film on the surface of modified loess particles is continuously dissolved, the large agglomerated particles are separated. The cementation between particles is weakened, the contact area is continuously reduced, the soil structure changes significantly, and the strength change of BML is more extensive than RL. With the increasing of W–D cycles, the influence of water migration on soil structure is weakened, and the soil particles and pores are rearranged, the particles are mainly point-to-point contact. However, due to the clay minerals in bentonite, the bond strength between the modified loess particles is greater than that of RL, so the shear strength of BML is higher than that of RL.

### Analysis of cohesion damage degree

The influence of W–D cycles on the internal friction angle of RL and BML is smaller than that of the cohesion. Therefore, in order to analyze the deterioration of RL and BML during W–D cycles, a continuous variable *λ* was introduced based on the cohesion, named the cohesion damage degree:

8 $$\left\{ \begin{gathered} \lambda_{1} = \frac{{\left| {c_{1} - c_{01} } \right|}}{{c_{01} }} \hfill \\ \lambda_{2} = \frac{{\left| {c_{2} - c_{02} } \right|}}{{c_{02} }} \hfill \\ \end{gathered} \right.$$

where *λ* = 0 corresponds to the non-damaged state, *λ* = 1 corresponds to the completely damaged state, 0 < *λ* < 1 corresponds to the different degree of cohesive damage state; *c*_01_ and *c*_02_ represent the cohesion of RL and BML samples in the initial state, respectively (kPa); *c*_1_ and *c*_2_ respectively represent the cohesion of RL and BML after W–D cycles (kPa).

According to the curve of cohesion of RL and BML under W–D cycles in Fig. 12a, it can be found that there are quadratic polynomial relationships and the exponential relationship between the cohesion *c* of RL and BML after experiencing different W–D cycles *N*:

9 $$\left\{ \begin{gathered} c_{1} = c_{01} + a_{1} N + b_{1} N^{2} \hfill \\ c_{2} = a_{2} * N^{{b_{2} }} \hfill \\ \end{gathered} \right.$$

where *a*_1_, *a*_2_, *b*_1_ and *b*_2_ are the fitted parameters, with values of − 10.1189, 46.0906, 0.5783 and − 0.3422, respectively.

Substituting Eq. (9) into Eq. (8), the relationship between the cohesion of RL and BML and the number of W–D cycles are obtained, respectively:

10 $$\left\{ \begin{gathered} \lambda_{1} = \frac{{\left| {N(a_{1} + b_{1} N)} \right|}}{{c_{01} }} \hfill \\ \lambda_{2} = \left| {1 - \frac{{a_{2} * N^{{b_{2} }} }}{{c_{02} }}} \right| \hfill \\ \end{gathered} \right.$$

where *λ*_1_ and *λ*_2_ are the cohesion damage degree of RL and BML after W–D cycles, respectively; *N* is the number of W–D cycles; *c*_01_ and *c*_02_ are the initial cohesion of RL and BML samples, respectively, with values of 58.68 kPa and 87.33 kPa; *a*_1_, *a*_2_, *b*_1_ and *b*_2_ are the fitted parameters, with values of − 10.1189, 46.0906, 0.5783 and − 0.3422, respectively.

In Eq. (10), *λ*_1_/*λ*_2_ can be obtained as Eq. (11), which reflects the quantitative relationship between the cohesion damage degree of RL and that of BML in the process of W–D cycles:

11 $$\frac{{\lambda_{1} }}{{\lambda_{2} }} = \left| {\frac{{N\left( {a_{1} + b_{1} N} \right)}}{{{\text{c}}_{{{02}}} - a_{2} * N^{{b_{2} }} }}} \right| * \frac{{c_{02} }}{{c_{01} }}$$

**Figure 14:**
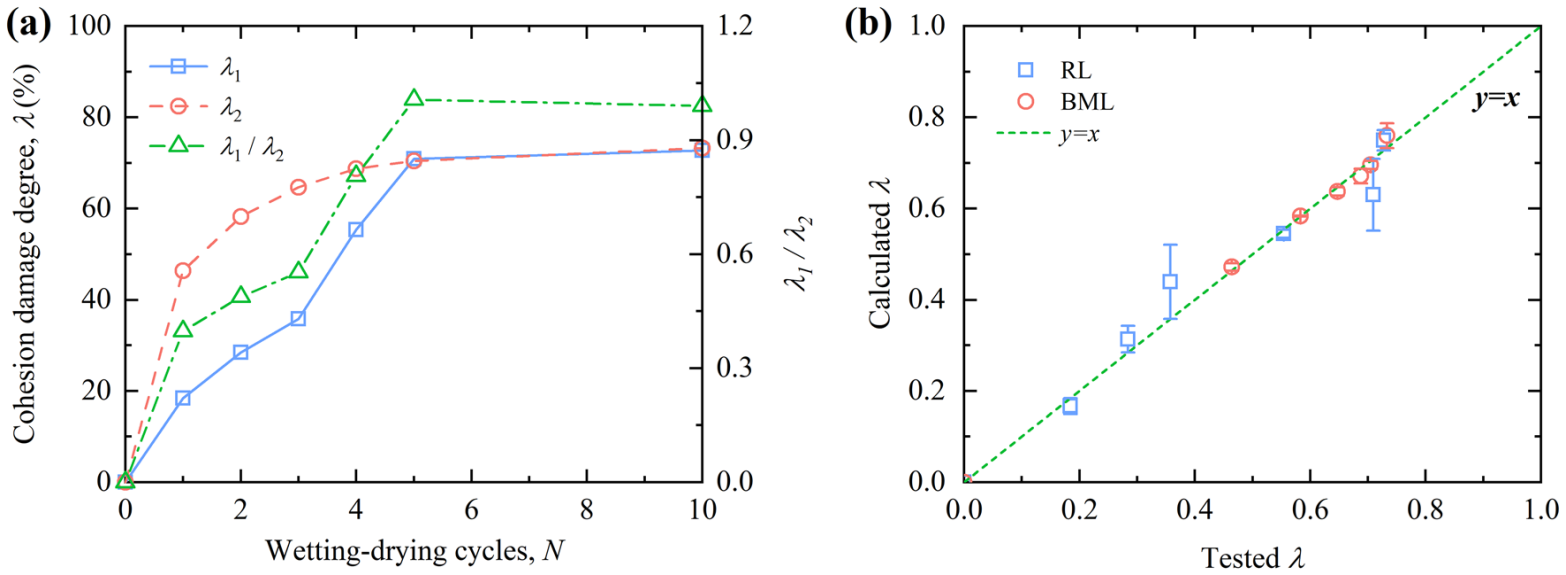
Comparison of cohesion damage degree and model results.

Figure 14a presents the curves of cohesion-based damage degree of RL and BML after different W–D cycles. The cohesion-based damage degree of RL and BML increases with the increase of W–D cycles and tends to be stable after five cycles. In the initial five W–D cycles, the damage degree of BML is always larger than that of RL, while the damage degrees for RL and BML approach to the same value after the fifth cycle. The bentonite inside the BML sample has strong swelling after absorbing water, while the suction due to capillary action is generated when it loses water, resulting in the tension stress field inside the soil. The tension stress concentration is generated in the local weak area of the soil sample. When the tension stress is larger than the tensile strength, the sample generates uneven cracks^34^. Therefore, the damage of the BML is always greater than that of the RL before the fifth W–D cycle.

The cohesion damage degree of RL and BML after W–D cycles was calculated by Eq. (8), and compared with the experimental values of cohesion damage degree. The comparison results are shown in Fig. 14b. It can be seen from the figure that the data points are distributed on both sides of the straight-line *y* = *x*, which indicates that the predicted value of the cohesive damage model is similar to the experimental value and that the model can better predict the damage regularity of the cohesion of RL and BML under the action of W–D cycles.

### Correlation analysis of shear strength and microstructure of loess

#### Multivariate linear stepwise regression method

The mechanical properties of loess and modified loess are not only related to their mineral composition, but also the degree of association of pores and particles. The expansion and development of surface cracks of loess under W–D cycles are closely related to the connection of internal particle pore structure. Therefore, to investigate the correlation between the strength characteristics of loess and microscopic particle pores, the regression equation between the shear strength parameters of loess and microscopic pore structure parameters was established by the multivariate linear stepwise regression analysis. The stepwise method involves introducing the independent variables into the model individually, conducting an F-test after each new independent variable is introduced, and performing a t-test on the selected independent variables singly, and removing the variables with insignificant changes from the equation individually. The expression of the model is as follows^35,36^:

12 $$Y = \beta_{0} + \beta_{1} X_{1} + \beta_{2} X_{2} + \cdots +  \beta_{i} X_{i}$$

where *β*_0_ is the regression constant; *β*_1_ is the partial regression coefficient of the independent variable *X*_1_; *β*_2_ is the partial regression coefficient of the independent variable *X*_2_; *β*_i_ is the partial regression coefficient of the independent variable *X*_i_; *i* is the number of independent variables. The results of partial regression coefficients and regression constants were obtained by data analysis.

In the multivariate linear stepwise regression analysis, the shear strength parameters were selected as the cohesion (*c*) and the internal friction angle (*φ*). The parameters of the micro-pore structure of loess were selected as equivalent diameter (*D*_*e*_), oblate degree (O_*d*_), pore area ratio (*n*), average form factor (*F*), probability entropy (*H*), fractal dimension (*D*_*f*_)^32,37^. The *D*_*e*_ is the average diameter of an equivalent circle equal to the pore area of the soil. The O_*d*_ is the ratio of the short axis and the long axis of the pore, the value is less than 1. The smaller the value, the pore tends to be elongated, otherwise, the closer to the round shape. The *n* indicates the ratio of the pore area to the total area within the same cross-section. The *F* characterizes the pore boundary roundness, and the value is in the range of (0,1), the pore shape is closer to round with the increase of *F*. The *H* is used to describe the directionality of the pore arrangement. The value of *H* varies between (0,1) and the smaller the *H*, the more pronounced the directionality of the pores. The *D*_*f*_ reflects the variation law of pore complexity with its area, and the larger the *D*_*f*_ reflects the more complex pore structure and the more dispersed pore distribution of the soil. The experimental results and the obtained parameters are shown in Table 3.

**Table 3:** Summary of strength and micro-pore structure parameters of loess.

Loess	Wetting–drying cycles	Strength parameters		Micro-pore structure parameters					
		*c*/kPa	*φ*/°	*D*_*e*_/μm	O_*d*_	*n*	*F*	*H*	*D*_*f*_
Remolded loess	0	58.68	21.42	4.5734	0.5850	29.60%	0.3728	0.9836	1.1971
	1	47.86	22.00	4.3102	0.6142	29.13%	0.3337	0.9917	1.2007
	2	42.00	20.86	4.7598	0.6074	36.65%	0.3529	0.9893	1.2377
	3	37.69	20.98	4.4649	0.6185	38.07%	0.3479	0.9920	1.2427
	4	29.17	24.45	4.5932	0.6102	40.48%	0.3412	0.9892	1.2328
	5	17.07	20.66	4.0995	0.6167	40.43%	0.3349	0.9947	1.2256
	10	16.00	20.75	4.7722	0.6236	42.53%	0.3298	0.9935	1.2195
Bentonite modified loess	0	87.33	31.50	3.7825	0.5909	16.57%	0.3842	0.9876	1.1626
	1	46.82	28.80	4.1319	0.584	20.08%	0.3916	0.9907	1.2211
	2	36.43	25.90	4.2751	0.5750	26.28%	0.3019	0.9913	1.2448
	3	30.83	20.20	4.3752	0.5865	30.86%	0.2982	0.9895	1.2557
	4	27.30	19.23	4.8088	0.5871	35.13%	0.3100	0.9838	1.2813
	5	25.81	18.00	4.5036	0.5919	38.07%	0.2880	0.9915	1.2727
	10	23.33	17.22	4.9375	0.6105	38.56%	0.3741	0.9894	1.2477

#### Results of regression analysis

The regression models of shear strength and micro-pore structure parameters were calculated by multivariate linear stepwise regression analysis through SPSS software and obtained as shown in Table 4. The cohesion of loess is negatively linearly correlated with the *n*, i.e., the cohesion decreases gradually with the increase of *n*. The *n*, *H*, and *D*_*f*_ can jointly explain 84% of the loess cohesion, indicating that the *n*, *H*, and *D*_*f*_ have a strong influence on the loess cohesion. The *c* is negatively correlated with *n*. The larger the pore area is, the easier for the particles to slide, and the bonding effect between the soil particles is weakened, leading to the reduction of the loess cohesion. The *c* is negatively correlated with *H*. With the increase of *H*, the pore orientation weakened, the pore direction distribution tended to be random, and the loess cohesion gradually decreased. The *c* is negatively correlated with *D*_*f*_. With the increase of *D*_*f*_, the complexity of pore distribution of different pore sizes and shapes in the plane increases, the more complex the pore structure of the soil, the more dispersed the pore distribution is, the easier the skeleton of soil particles will be destroyed, and the loess cohesion will be smaller.

**Table 4:** Regression analysis of strength and micro-pore structure parameters of loess.

		Regression model	*R*^2^	*F*	*P*
*c*	Model 1	*c* = 102.061 − 195.169 *n*	0.65	25.238	< 0.001
	Model 2	*c* = 394.346 − 142.838 *n* − 251.362 *D*_*f*_	0.77	22.257	< 0.001
	Model 3	*c* = 2087.196 − 115.357 *n* − 285.656 *D*_*f*_ − 1676.723 *H*	0.84	22.976	< 0.001
*φ*		*φ* = 35.657 − 40.486 *n*	0.60	20.368	< 0.001

The *φ* is linearly correlated with the *n*, and the correlation coefficient of the regression equation is 0.6. The *φ* is negatively correlated with the *n*, i.e., the *φ* decreases with the increase of the *n*. The larger *n* is, the more pores in the unit area, the bonding between soil particles is weakened, the easier the sliding between particles, and the internal friction angle decreases. It can be seen that the *n* is one of the factors affecting the *φ* of loess.

**Figure 15:**
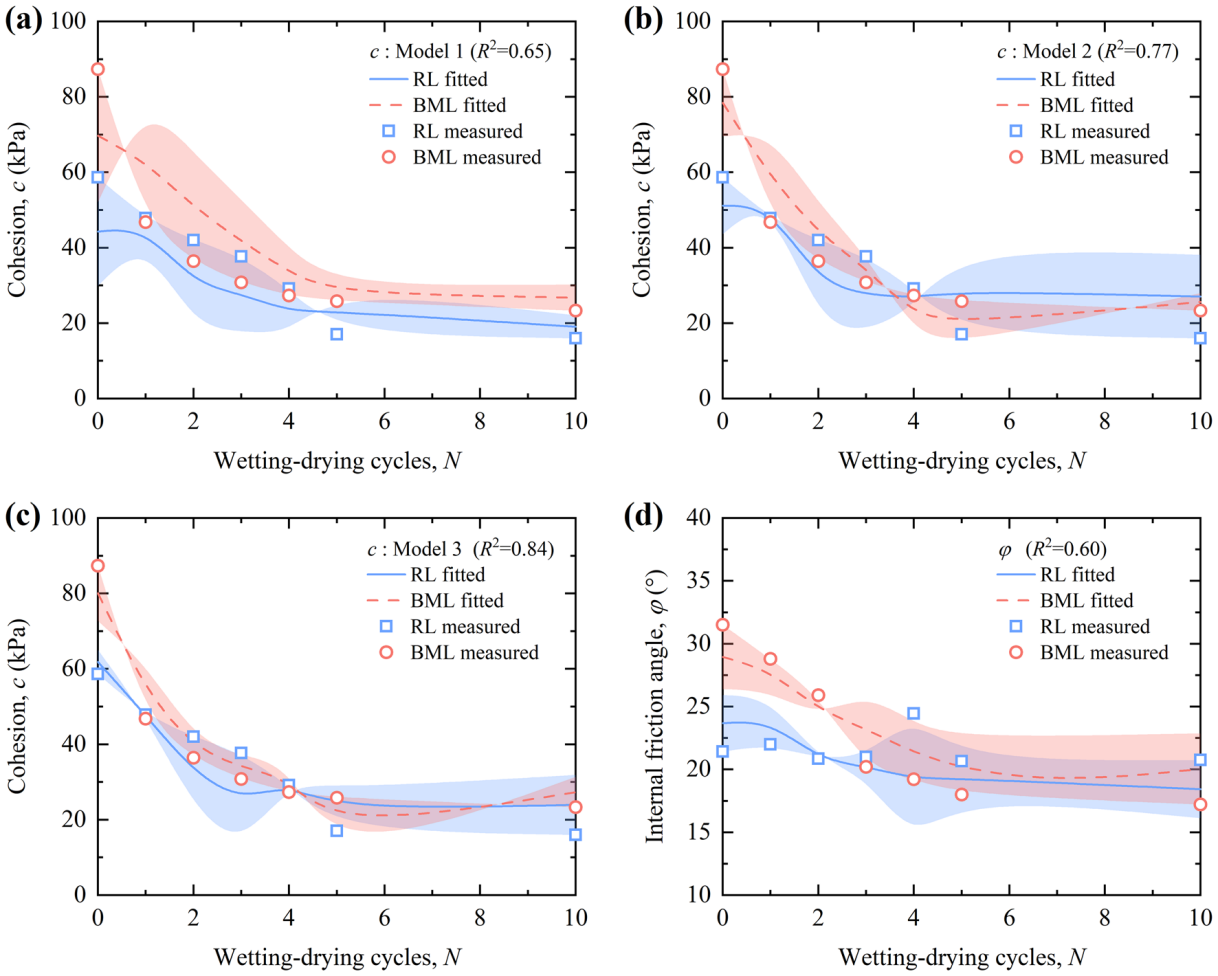
Comparison of measured and fitted values of shear strength parameters.

Figure 15a–c illustrate the comparisons between the fit results of the stepwise regression equations and the measured values of the cohesion of RL and BML. From the figures, as the independent variables in the regression equation increase, the range of error bands gradually decreases and the correlation coefficients of the regression models increase subsequently. When the regression model contains *n*, *H*, and *D*_*f*_, the fitted correlation coefficient *R*^2^ is 0.84, and the fitted value of cohesion has a high linear correlation with the measured value, indicating that the fitted regression model 3 can be used to predict the cohesion of loess. Figure 15d shows the comparison between the measured and fitted values of the internal friction angle. The fitted values of the internal friction angle fit well with the measured, and both have similar decay laws with the increase of wetting–drying cycles. It is indicated that the regression equation between macro-mechanical parameters and microstructural parameters established by multivariate linear stepwise regression method can well reflect the influence law of loess microstructure on macro-mechanical strength.

## Discussions

### Microstructure evolution of BML under W–D cycles

In the process of W–D cycles, the soil mechanical properties are very complex, which are affected by many factors, such as soil water retention characteristics, mineral composition, W–D cycle stage, microstructural characteristics, and cracking intensity^38^. Ni et al.^18^ considered that the reasons for the changes of loess particle microstructure under W–D cycles were that the dissolution and recrystallization of soluble salts at different water contents affected the size and shape of the particle’s skeleton, and the clay cementation was affected by swelling and suction, and caused the arrangement of loess particles. The W–D cycle is essentially the process of water migration inside the soil. In this process, the pores in the soil are used as migration channels. The water continuously washes the primary pores, causing the dissolution of some fine particles and part of clay minerals on the surface, making the primary small and medium pores transition to large pores, weakening the soil structure and reducing the soil strength. The essence of the microstructure change of BML under the action of the W–D cycles is that the cementation strength and double electron layer destruction of the clay minerals such as montmorillonite on the surface of the modified loess particles make some cementation aggregates separate into small particles, the particles are rearranged, and the microstructure of the soil gradually be destroyed and forms a new stable structure. As the W–D cycles continue, the pore structure almost does not change, and the soil strength reaches stability.

### Multiscale comparison of damage regulation of loess under W–D cycles

The deformation of soil can be divided into macroscopic deformation and microscopic deformation, and the macro characteristics such as strength are the reflections of the microscopic structure of the soil. The combination of macro strength test and micropore structure observation can better observe and explain the deformation failure regularity of loess.

**Figure 16:**
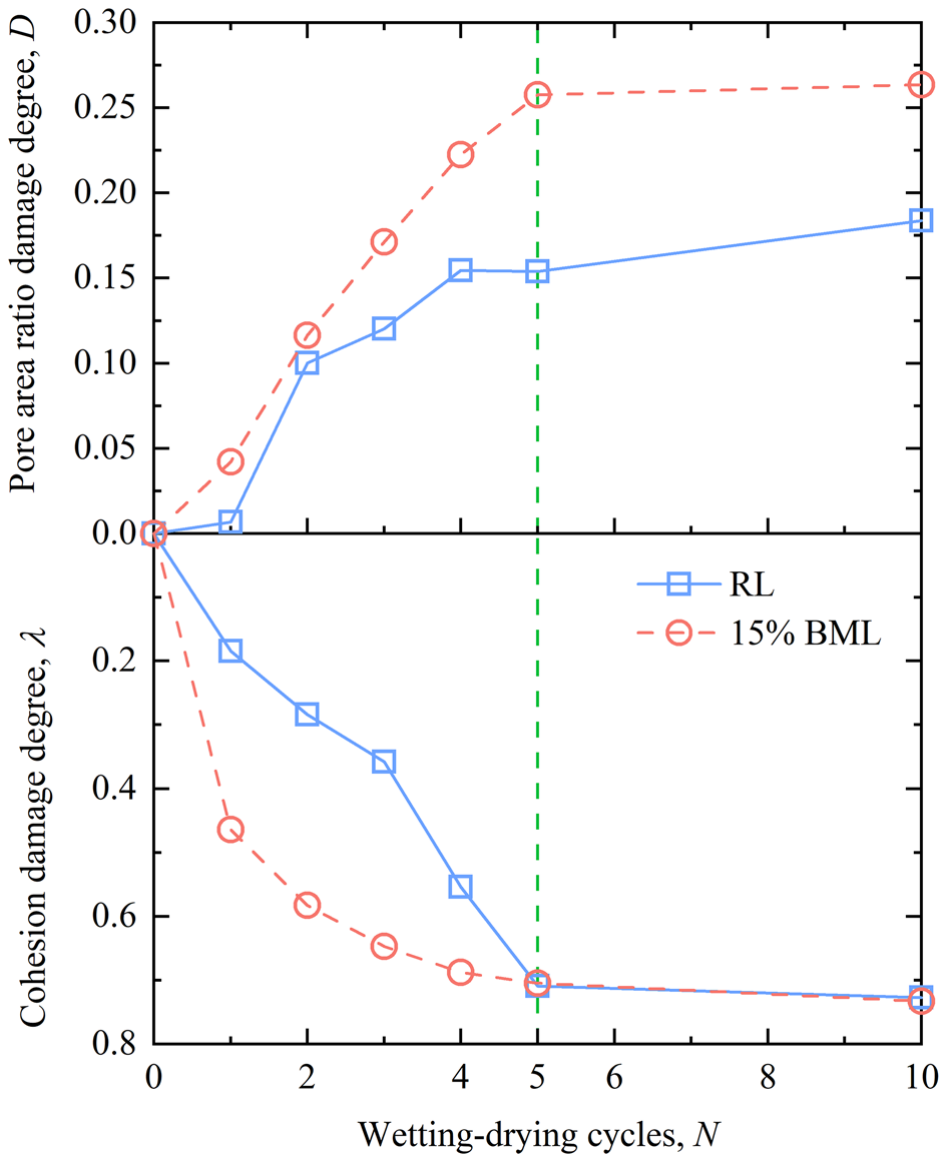
Comparison of damage degree of loess under W–D cycles.

Figure 16 illustrates the curve of damage degree of RL and BML under different W–D cycles. The variation of damage degree of soil pore area ratio and cohesion is consistent under the action of W–D cycles. The damage degree of samples increases with the increase of W–D cycles; after five W–D cycles, the damage degree of the sample tends to be stable. It is revealed that the W–D cycles have a decelerating degradation effect on the loess before and after bentonite modification. The damage of soil is reflected by the cohesion and micro-pore area ratio, which have macro and micro consistency.

## Conclusions

This paper took RL and BML as study objects, through SEM tests and direct shear tests to research the damaged relationship between microstructure and macroscopic strength of samples under different W–D cycles. The main conclusions are as follows:

1. The bentonite absorbs water and swells to fill the macropores in the framework of loess particles, and the macropores decrease while the mesopores and micropores increase. The particle contact of RL is mainly pointing contact, while the BML is surface contact. The microstructure of BML is more stable than RL under W–D cycles.
2. With the increase of W–D cycles, the cement structure and diffusion electric double layers of the loess are destroyed, resulting in the loss of the particle arrangement of RL and BML, and the pore area ratio and pore fractal dimension increase gradually. After five W–D cycles, the particles are rearranged until the soils reach a new stable structure, the pore area ratio and fractal dimension are increasingly stable, but the pore area ratio of BML is smaller than RL, the microstructure is more complex, and the pore fractal dimension is more significant.
3. The cohesion of RL and BML all decreases under the W–D cycles, but the decay rates of BML are larger than RL. The internal friction angle of RL has no noticeable change under W–D cycles, but the internal friction angle of BML decreases with the increase of W–D cycles.
4. The regression equation between macro-mechanical parameters and microstructural parameters established by multivariate linear stepwise regression method can well reflect the influence law of loess microstructure on macro-mechanical strength.
5. Based on the changes of pore area ratio and cohesion of samples during the W–D cycles, the continuous damage variables about pore area ratio and cohesion are introduced respectively, establishing damage degree models of pore area ratio and cohesion, which can better predict the damage regulation of pore area ratio and cohesion of RL and BML during the W–D cycles.

## Data Availability

The data used to support the findings of this study are included within the article.
